# Deviation from physiologically appropriate oxygen levels alters proliferation, cytokine production and proximal antigen receptor signalling in CD4+ memory T cells

**DOI:** 10.3389/fimmu.2026.1833034

**Published:** 2026-05-26

**Authors:** Elizabeth Clay, Graham R. Wallace, Stephen P. Young

**Affiliations:** 1 Rheumatology Research Group Centre for Translational Inflammation Research, College of Medical and Dental Sciences, School of Immunity and Infection, University of Birmingham, Birmingham, United Kingdom; 2 Academic Unit for Ophthalmology, Centre for Translational Inflammation Research, College of Medical and Dental Sciences, School of Immunity and Infection, University of Birmingham, Birmingham, United Kingdom

**Keywords:** CD4+ memory T cell, hypoxia, physiological oxygen levels, redox, T cell signaling, *in vitro* culture

## Abstract

**Background:**

Immune cells must adapt to highly variable oxygen levels encountered across different tissues of the body. Under inflammatory conditions, oxygen levels may reach further extremes, for example healthy synovial joints display oxygen levels of 6-10%, which drop to <5% in rheumatoid arthritis. In contrast, most *in vitro* experiments are performed under atmospheric oxygen (~21%), raising concerns that experimental conditions do not accurately reflect the physiological microenvironment of the disease being modelled.

**Aim:**

To explore the role of oxygen levels (21%, 8.5%, 3% and 1% oxygen) on human memory CD4+ T cell function.

**Results:**

At oxygen levels reflective of physiologically healthy conditions we observed increased proliferative capacity and reduced pro-inflammatory cytokine production of CD4+ memory T cells. Altered patterns were observed in both hyperoxic and hypoxic conditions. A critical component of TCR proximal signaling, Lck phosphorylation, was altered in resting T cells equilibrated to varying oxygen levels. The highest ‘activated’ state was seen at physiologically healthy oxygen levels, suggesting prior oxygen exposure can determine subsequent signaling responses.

**Conclusion:**

We conclude that environmental oxygen levels significantly influence CD4+ memory T cell responses, with implications for their function in inflammatory sites *in vivo.* These differences need to be taken into account when designing or interpreting *in vitro* experiments, as well as harnessing T cells therapeutically.

## Introduction

Lymphocytes are exposed to a range of oxygen levels as they circulate throughout the body. However, despite the development of hypoxia-responsive CAR T cells (1), and the acknowledged variation in physiologic oxygen levels (2), characterisation of T cell responses across the spectrum of oxygen levels encountered in human tissue remains lacking.

Secondary lymphoid organs are hypoxic environments, with the thymus and lymph nodes exhibiting oxygen levels around ~0.5-1% oxygen (3–8). Distance from blood vessels contributes to oxygen exposure and this appears to have relevance to the distribution of immune cells. For example, in tumours T cells cluster around vessels and display a less active phenotype when further from vasculature (7, 9). Peripheral blood is better oxygenated, but levels vary between arteriolar and venous circulation (10). In the periphery, tissues show gradients of oxygen in a site-specific manner – for example the intestinal lamina propria exhibits oxygen levels of around 7%, but the intestinal lumen itself is almost devoid of oxygen (2, 11).

Under chronic inflammatory conditions, oxygen levels are typically low compared to healthy tissues. The synovial joint in rheumatoid arthritis provides a clear example: oxygen levels measured in healthy synovial fluids is range between 6% to 10% (12–14), whereas under inflamed conditions oxygen levels in synovial fluid and tissue fall to around 3% (12, 13, 15–17) or even <1% in severe cases (13, 15). Indeed, oxygen levels inversely correlate with macroscopic synovitis, suggesting that more severe disease is associated with poorer tissue oxygenation (15). Increased oxygen consumption is driven by proliferating synovial fibroblasts and macrophages (18) as well as substantial infiltration by immune cells including neutrophils, plasma cells and both CD8+ and CD4+ T cells (19). Despite genetic evidence suggesting they play an important role in RA pathology (20, 21), synovial CD4+ T cell function appears depressed (22), potentially reflecting adaptation to, or suppression by, exposure to a chronically hypoxic environment (23, 24). It is therefore important to understand how oxygen may affect cellular function.

Much of the research into the effects of oxygen deprivation on T cell behaviour has focused on comparing 1% oxygen to culturing at atmospheric oxygen (~21%), omitting a comparison with other more physiologically relevant, and healthy, oxygen levels. Indeed, 21% oxygen is commonly referred to as ‘normoxia’ in the literature; however, there is nothing ‘normal’ about this oxygen level for *ex vivo* cells (25). At both hyperoxic and hypoxic conditions of 21% and 1% oxygen, respectively, cells are expected to be under increased oxidative stress. At 21% oxygen this increase in ROS includes, but is not limited to, the action of NOX enzymes. Furthermore, an excess in molecular oxygen can increase superoxide formation from the electron transport chain (26, 27). At 1% oxygen an increase in oxidative stress is due to the strain mitochondria are placed under when oxygen is lacking (28–30).

Hence, lymphocytes are exposed to a wide range of oxygen levels physiologically but never experience the high levels commonly used in *in vitro* experiments. We set out to determine how varying oxygen levels affect CD4+ memory T cell function. Oxygen conditions were selected to reflect those measured in healthy and inflamed synovial tissue, which are also representative of gradients observed across other organs (2). Importantly, media and cells were pre-equilibrated to the specified oxygen level before stimulation.

## Materials and methods

### CD4+ memory T cell isolation

Peripheral Blood Mononuclear cells (PBMCs) were obtained from buffy coats from the National Blood Service or from informed and consented healthy controls under local NHS ethics project code 12/WM/0077. Ficoll-paque density gradient centrifugation was used to obtain PBMCs and human Memory CD4+ T cell isolation kit, Miltenyi (Bisley, UK), was used to isolate CD4+ CD45RO+ memory cells. Double isolations were performed to increase purity and cells were stored at -80˚C before use.

### CD4+ memory T cell culture at varying oxygen levels

Effector memory cell populations were plated at 1x10^6^ viable cells/ml in RPMI-1640 culture medium (Sigma Aldrich,St. Louis, MO, USA);/10% FBS (Labtech, East Sussex, UK)/1% L-Glutamine-Penicillin-Streptomycin solution (Sigma-Aldrich) and placed on ice for transfer into either a normal CO_2_ incubator (Function Line, Thermo scientific) or H35 Hypoxystation (Don Whitley, West Yorkshire, UK). Both incubators were maintained at 37˚ C and 5% CO_2_, and in the hypoxystation oxygen levels were balanced using nitrogen gas. All cultures were equilibrated to their designated oxygen level for 24 hours before activation. For assessment of Lck phosphorylation, cells were fixed at the oxygen level they had been acclimatised to. When stated, cells were stimulated with plate-bound anti-CD3/anti-CD28 (2μg/ml anti-CD3//5μg/ml anti-CD28). Prior to removal from specified oxygen environments, cells were placed on ice at the oxygen level they had been stimulated at for 4–5 minutes, to limit the effects of reperfusion injury at 37˚C.

### CD4+ memory T cell CFSE staining and calculation of proliferative index

To assess proliferation, cells were incubated with 1μM carboxyfluorescein diacetate succinimidyl ester (CFSE) diluted in phosphate buffered saline (PBS) (Invitrogen,Paisley, UK) for 15 minutes at 37˚ C before being quenched with ice-cold culture medium. Cells were washed, equilibrated and stimulated as described above for four days before assessing proliferation on a CyAn ADP Analyser, (Beckman Coulter,High Wycombe, UK). Stimulated cells were gated on for assessment of proliferation (**Supplementary Figure 1**). Proliferative Index was calculated by dividing the number of cells in each division gate by the number of times those cells had divided and summing cells in all gates. We then divided the actual total number of cells in gates by this number to estimate an index of overall proliferation, similar to a widely used procedure (FlowJo Manual).

### Antibodies and reagents

Purified anti-CD3 and anti-CD28 from Immunotools (Friesoythe, Germany) were used for plate-bound stimulation. Antibodies were obtained from Biolegend (London, UK) including anti-human CD69 APC Cy7, APC/Cy7 Mouse IgG1 k isotype control, Pacific Blue anti-human IFNγ, Pacific Blue Mouse IgG1 k isotype control, PerCP/Cy5.5 anti-T-bet, PerCP/Cy5.5 mouse IgG2b l isotype control, PE anti-human CD45RO and PE mouse IgG2a k isotype control. Anti-pLck(Tyr394) unconjugated polyclonal rabbit antibody was purchased from Santa Cruz Biotechnology (Dallas, TX, US). Goat anti-rabbit IgG (H+L) FITC was obtained from Southern Biotech (Birmingham, AL, USA).

### Flow cytometry

Surface staining was carried out using antibody cocktails diluted in 2% BSA/PBS. Intracellular cytokine staining of cells was performed using 2μg/ml Brefaldin A Ready Made Solution (Sigma-Aldrich) 3 hours before the end of the indicated stimulation period, with fixation and permeabilization using Invitrogen Fixation Medium and Permeabilisation Medium. A CyAn ADP Analyser was used to determine expression of markers. Countbright Absolute Counting Beads (Life Technologies (Paisley, UK) were used to maintain consistent mean fluorescence intensity (MFI) values over time. For compensation, BD CompBeads Anti-mouse Ig, k/Negative Control (FBS) Compensation Particles Set, and Anti-Rat IgG/Negative Control (FBS) Compensation Particles Set were used.

### ELISA

Supernatants were collected from 48 hour stimulated samples (with 3 hours Brefaldin A treatment at the end of the stimulation period) and stored at -80˚C until required. eBioscience Ready-SET-Go! ELISA kits were used to assess supernatant cytokine levels for IFNγ, TNFα, IL-17A, IL-10, IL-4, and IL-5.

### Statistical analysis

For statistical analysis, the Mann Whitney U test was used as a non-parametric, unpaired test. Pairing was not possible as the healthy human donor cell numbers were variable and so not every oxygen level could be assessed for each donor. GraphPad Prism software was used for the generation of graphs and performing statistical analysis. Adobe Illustrator was used for figure generation.

## Results

Four different oxygen levels were selected to represent those experienced by T cells either physiologically or during *in vitro* experimentation. Specifically, we included: 21% oxygen (atmospheric oxygen) to represent the condition used in most *in vitro* experiments; 8.5% oxygen as the average level present in healthy tissues including the healthy joint; 3% oxygen as the average level found in the inflamed joint (12, 13, 15–17); and 1% oxygen as an example of the level observed in severely inflamed joints (15). Cells were equilibrated to the selected oxygen level for 24 hours before stimulation in agreement with the observation of cytosol oxygen equilibration observed by Chapple et al. (31).

### CD4+ memory T cell proliferation is increased at physiologically healthy oxygen levels

**Figure 1:**
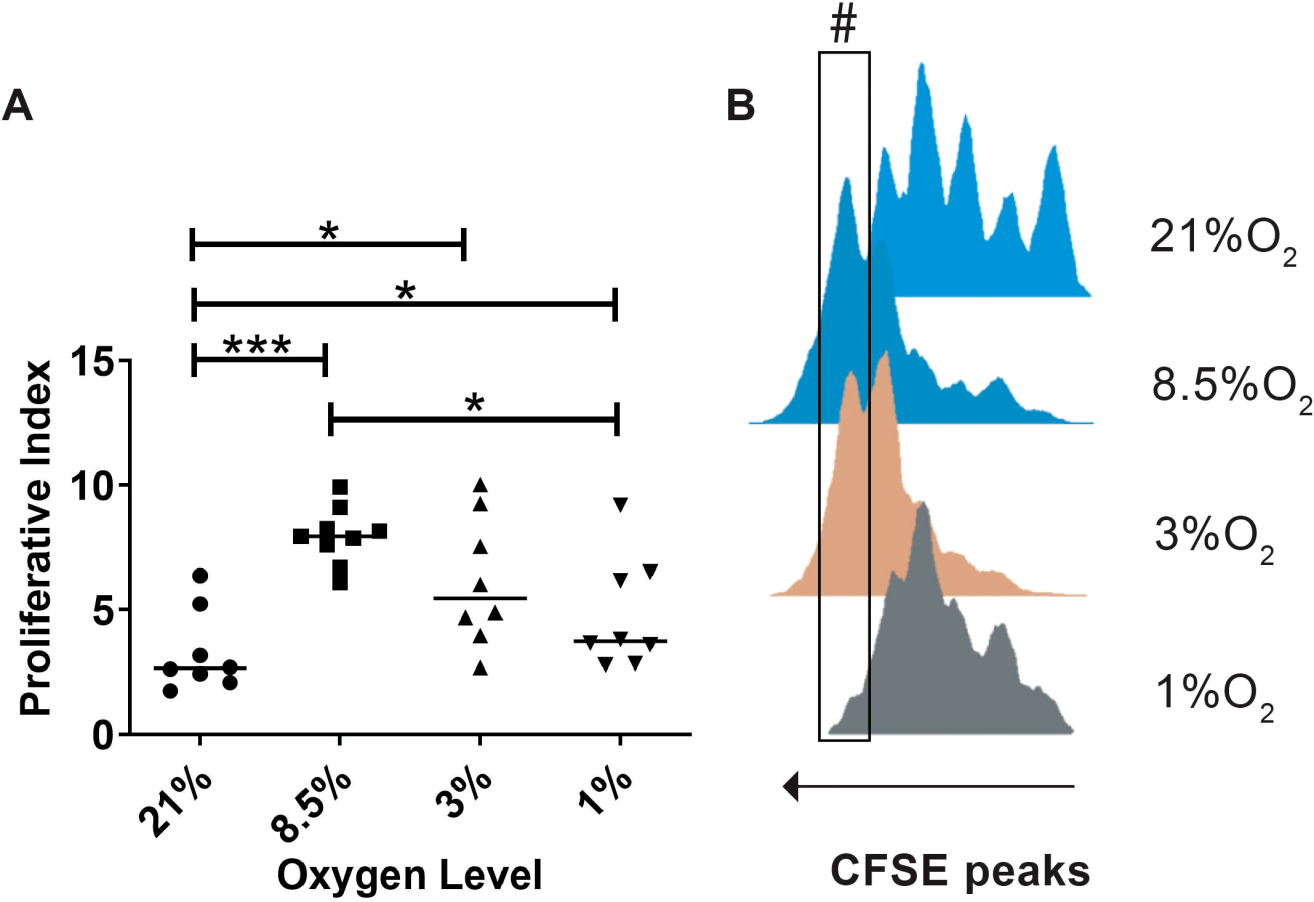
CD4+ memory T cell proliferation is increased at physiologically healthy oxygen levels. CD4+ memory T cells were stained with 1μM CFSE and stimulated after 24 hours equilibration to the stated oxygen level. Proliferation was determined after four days stimulation. **(A)** The proliferative index of CD4+ memory T cells stimulated at each of the oxygen levels investigated. **(B)** Example CFSE plots after four days stimulation at different oxygen levels. The black box shows 5 cell divisions. Data from 8 healthy donors. Mann Whitney U statistical test was used and median values are shown. *p<0.05, ***p<0.001, #p<0.05 8.5% vs 1% and p<0.005 8.5 vs 21%.

We initially investigated the effects of environmental oxygen on CD4+ memory T cell proliferation after four days of stimulation. A marked increase in proliferation was observed in CD4+ memory T cells stimulated in 8.5% oxygen levels compared to all other oxygen levels investigated (**Figure 1**) with a significantly higher proliferative index (**Figure 1A**). Furthermore, at 8.5% oxygen, an increase in the number of cells reaching five divisions was observed (**Figure 1B**) (p< 0.05 for 8.5% oxygen vs 1% oxygen, and p <0.005 for 8.5% oxygen vs 21% oxygen). In contrast, stimulation at 21% oxygen revealed a stark reduction in CD4+ memory T cell proliferation compared to all other oxygen levels in both proliferative index and number of cells dividing five times (**Figures 1A, B**).

### Cytokine production in CD4+ memory T cells is altered after stimulation at different oxygen levels

**Figure 2:**
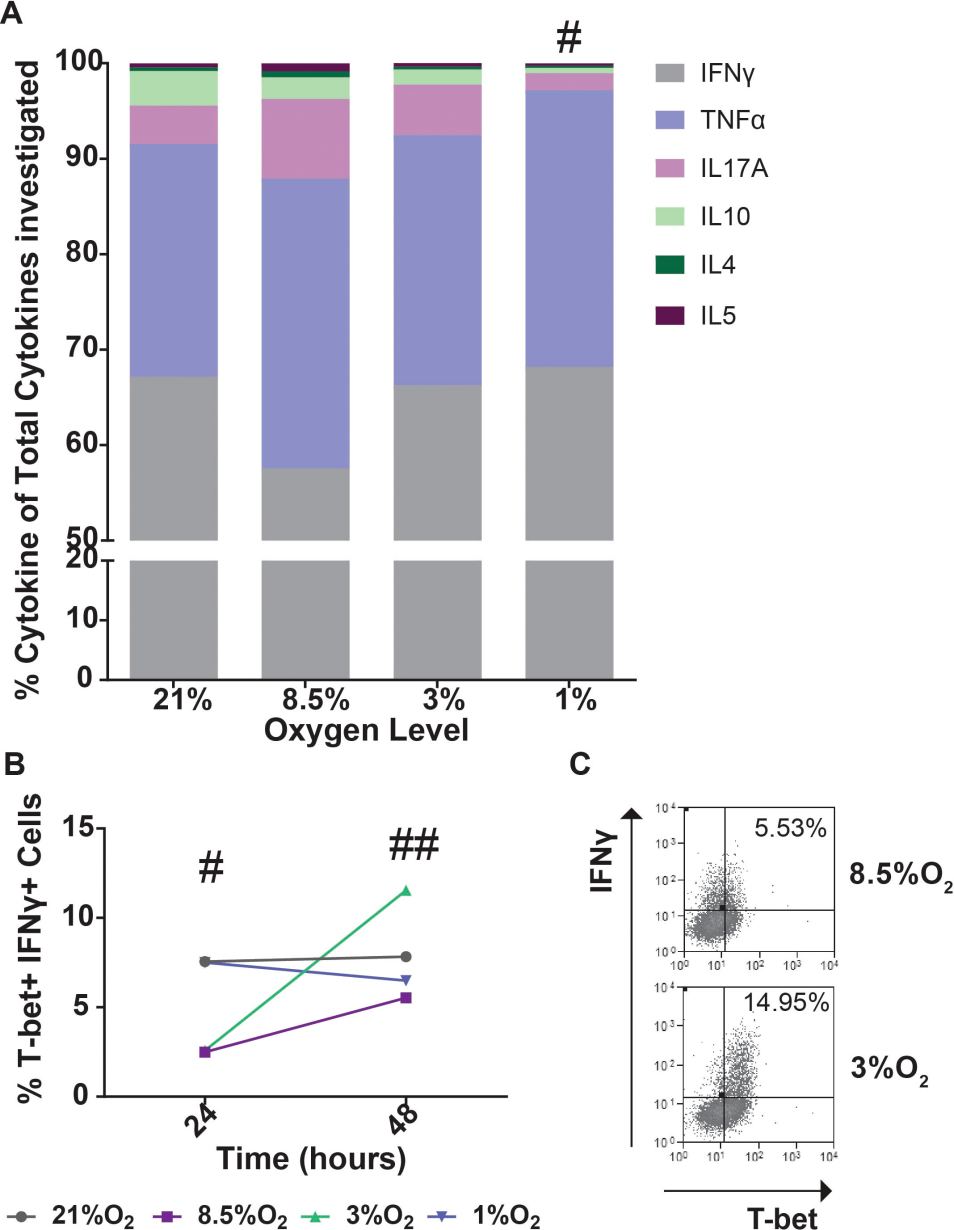
CD4+ memory T cells have more of a pro-inflammatory cytokine profile at low oxygen levels. Flow cytometry and ELISA were performed on CD4+ memory T cells and their supernatants after anti-CD3/CD28 stimulation (with the last 3 hours brefeldin A treated concurrent with subsequent intracellular staining). Cells were equilibrated to the stated oxygen level for 24 hours before stimulation. **(A)** ELISA of cytokines after 48 hours stimulation. Six cytokines were chosen for investigation: IFNγ; TNFα; IL-17A; IL-4; IL-10; IL-5. The overall contribution of the six cytokines to the cytokine population was assessed as a percentage of the overall cytokines investigated. #, p<0.001 for cumulative percentages of pro-inflammatory cytokine IFNγ, TNFα and IL-17A, compared to 8.5% oxygen, using Mann Whitney U statistical analysis. n= 7–10 donors per oxygen level. **(B)** Flow cytometry of T-bet cell surface marker and intracellular IFNγ expression. The percentage of Tbet+IFNγ+ cells was assessed after 24 hours and 48 hours stimulation. #p<0.05 1% compared to 8.5% oxygen, and p<0.005 21% compared with 8.5% oxygen at 24 hours. ##p<0.05 3% compared to 8.5% oxygen at 48 hours Mann Whitney U statistical test was used and median values are shown. A minimum of six donors were assessed at each oxygen level. **(C)** Representative plots are shown of plots from 8.5% and 3% cultures after 48 hours stimulation.

Cytokine production was assessed by ELISA, with quantification of three cytokines central to pro-inflammatory immune responses in multiple autoimmune diseases (IFNγ, TNFα and IL-17A) and three cytokines associated with anti-inflammatory and humoral immunity (IL-10, IL-4, and IL-5) (**Figure 2A**). We observed that pro-inflammatory cytokines were lowest under 8.5% and 3% oxygen, and highest at 1% oxygen (p<0.001 for cumulative percentages of pro-inflammatory cytokine IFNγ, TNFα and IL-17A, 1% oxygen compared to 8.5% oxygen).

Alterations in patterns of cytokine production over time were also shown to differ at the oxygen levels explored. After 24 and 48 hours stimulation at the various oxygen levels, intracellular and surface marker staining was performed to further assess cytokine production and T cell skewing, assessed using flow cytometry. Furthering the observations from the ELISA, reduced numbers of Th1-like T-bet+IFNγ+ cells were observed at 8.5% oxygen after both 24 and 48 hours stimulation (p<0.005 21% compared with 8.5% oxygen and p<0.05 1% compared to 8.5% oxygen at 24 hours. p<0.05 3% compared to 8.5% oxygen at 48 hours) (**Figures 2B, C**). For cells cultured at 21% oxygen, accumulation of T-bet+IFNγ+ cells was diminished compared to 3% oxygen, and in 1% oxygen levels dropped.

### CD4+ memory T cells reveal alterations in proximal T cell signaling and expression of activation markers at different oxygen levels

**Figure 3:**
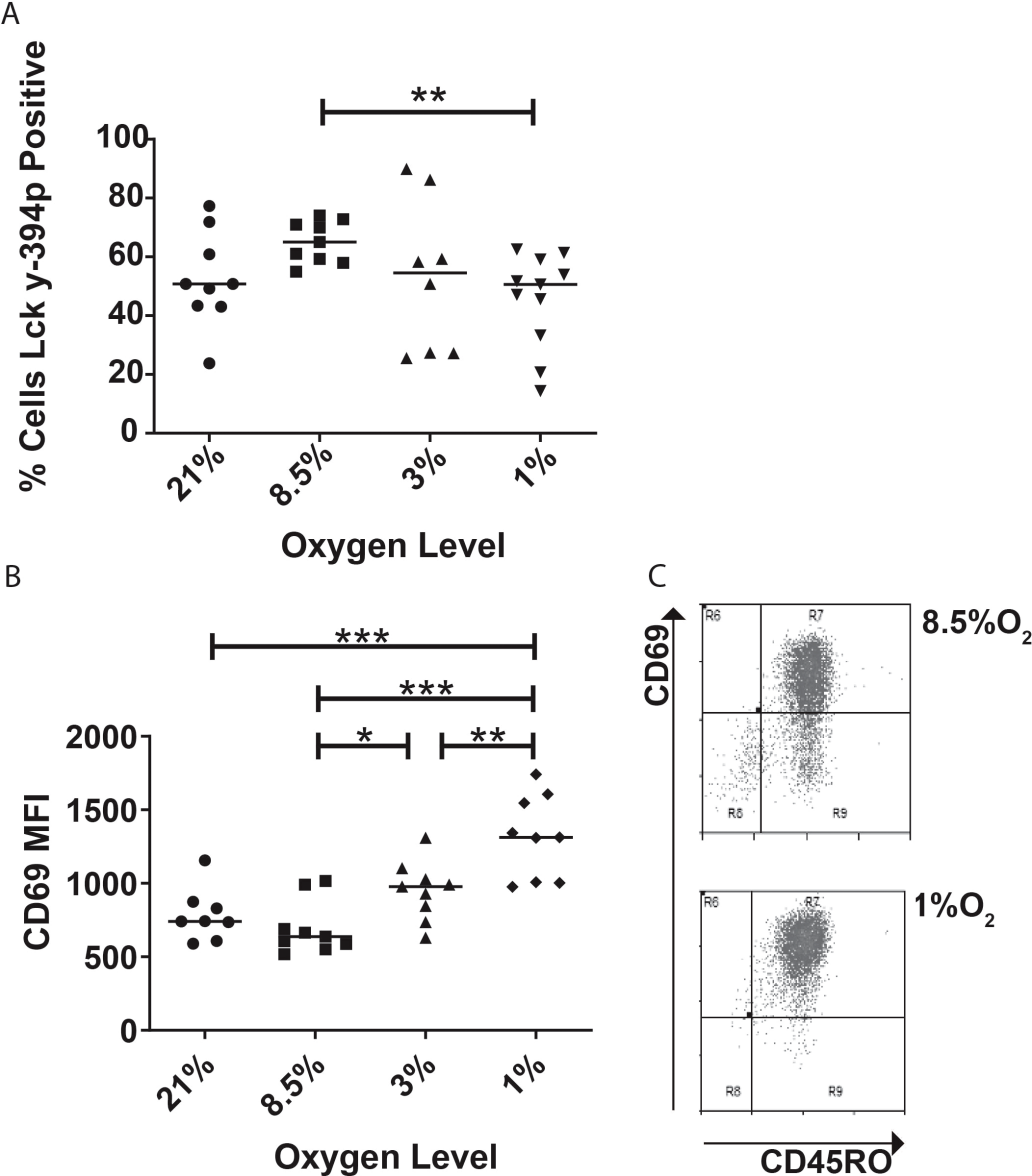
CD4+ memory T cells reveal alterations in proximal signaling apparatus and early activation markers at different oxygen levels. **(A)** CD4+ memory T cells were equilibrated at 21%, 8.5%, 3% and 1% oxygen for 24 hours before being fixed with 4% paraformaldehyde directly at the oxygen level studied. Cells (unstimulated) were stained for phosphorylation at Tyr394 on Lck and assessed by flow cytometry. Percentage positive cells were determined by comparison with isotype controls. A Mann Whitney U statistical test was used for the analysis and median values are shown in the data of a minimum of 8 donors. **p< 0.005. **(B)** Cells were stained for expression of CD69 and assessed by flow cytometry. The MFI of CD69 expression was determined after 48 hours stimulation at the oxygen levels stated. *p< 0.05, **p< 0.01, ***p<0.001. **(C)** Example flow cytometry plots showing CD45RO versus CD69 for cells cultured at 8.5% and 1% oxygen are shown, representative of data in **(B)**.

Alterations in proliferation suggested that equilibration at different oxygen levels prior to stimulation may influence T cell receptor (TCR) proximal signaling and early activation markers. Proximal T cell signaling is a complex and highly regulated process; Lck is a tyrosine kinase involved in the phosphorylation of key residues on the TCR apparatus, allowing proteins to dock and initiate downstream signaling. Phosphorylation at tyrosine 394 activates the Lck molecules, allowing it to phosphorylate downstream targets and initiate TCR signaling. It has been highlighted that the phosphorylation state of Lck in resting cells is important for subsequent signaling (32, 33). To investigate whether environmental oxygen levels influence proximal TCR signaling prior to stimulation, we assessed the phosphorylation of Lck at the activatory tyrosine residue 394 in resting CD4+ memory T cells after 24 hours equilibration at the various oxygen levels investigated. Phosphorylation of Lck on Tyr394 was higher at 8.5% oxygen compared to the alternative oxygen levels investigated, with significant reduction in phosphorylation observed at 1% oxygen. (**Figure 3A**). Further to this, staining for CD69, an early activation marker, revealed an inverse relationship between oxygen level and CD69 expression, with a significant increase in expression when cells were stimulated at 1% oxygen compared to higher oxygen levels (21% and 8.5%) after 48 hours stimulation (**Figure 3B**). Stimulation at 3% oxygen revealed intermediate levels of CD69 expression, highlighting CD69 expression levels may reflect the extent of oxygen depletion in the environment (**Figure 3B**). Interestingly, treating cells with the anti-oxidant *N*-acetyl cysteine (NAC) at 1% oxygen increased Lck phosphorylation. Furthermore, NAC treatment at 1% oxygen reduced CD69 expression similar to that seen at 8.5% oxygen (**Supplementary Figure 2**). This therefore highlights the role redox plays in the phenotype of CD4+ memory T cells in hypoxia.

## Discussion

CD4+ T cells are exposed to a wide range of oxygen levels as they circulate but *in vivo* will never experience the atmospheric oxygen levels commonly used for many *in vitro* experiments. Here, we exposed CD4+ memory T cells to 21%, 8.5%, 3% and 1% oxygen, equilibrating them before stimulation, and assessed their proliferation and activation. CD4+ memory T cells were selected for study as they are prevalent in chronic inflammatory environments such as the rheumatoid joint and are more likely to be re-activated in the tissue, whereas their naïve counterparts are initially activated by dendritic cells in hypoxic lymph nodes (34). They are also known to differ from the naïve counterparts in terms of their mitochondrial and metabolic resilience to hypoxia (35).

After culture at 21% oxygen, CD4+ memory T cells showed greatly reduced proliferation compared to those cultured at oxygen levels deemed more physiologically healthy (8.5%), where proliferation was at its peak. This agrees with previous studies also showing an increase in long-term proliferation at the physiologically healthy oxygen level of 5% oxygen (36–39). However, this reduction in proliferation at 21% oxygen contrasts with that seen in mixed lymphocyte populations, including mixtures of naïve and memory CD4+ cells (40–43). Reduced proliferation was also observed in cells exposed to hypoxia (1% oxygen). Previously, *in vitro* culture of CD4+ T cells at oxygen levels below 5% have been shown to depress proliferation (9, 41, 44–48). Interestingly, proliferation following stimulation by methods that bypass the TCR may be unaffected by lower oxygen levels (44) which would support our observation that alterations in proximal TCR regulation may underly the effects of different oxygen levels on T cell responses.

We therefore have shown a proliferative deficit of CD4+ memory T cells at both extremes of environmental oxygen levels, highlighting that more physiologically healthy oxygen levels may be a better and more suitable comparison for more extreme hypoxia, such as that seen in cancer and autoimmune settings. A potential explanation may be due to the oxidative environment of both hyperoxic and hypoxic cultures. Cellular exposure to hypoxia is known to result in increased released of ROS from both the mitochondrial electron transport chain (28–30) and extra-mitochondrial sources (49), and this has been specifically observed in stimulated CD4+ T cells (41). In addition, mixed T lymphocyte populations cultured at 21% oxygen have been shown to have reduced glutathione compared to physiologically healthy oxygen levels (5%) (40). As the redox environment has been previously implicated in negatively regulating T cell proliferation (41, 50–52), it is possible that when a redox imbalance occurs in both hyperoxic or hypoxic conditions, it may result in reduced long-term proliferation.

Cytokine production is also thought to be affected by environmental oxygen levels but reports again vary. Upregulation of cytokine secretion including IFNγ and IL-2 has been observed in response to culture in oxygen levels below 5% (41, 53–55) while some report a reduction in the mRNA and secreted protein of these two particular cytokines in response to oxygen levels lower than 2.5% (7, 43, 56). This may be in part due to the stabilisation of the hypoxia-responsive transcription factor, hypoxia-inducible factor-1alpha (HIF-1α) at low oxygen levels (57) and additionally may reflect what is observed in settings akin to high-altitude hypoxia, where, in a model of colitis, IFNγ production was increased in mice exposed to oxygen levels mimicking high-altitude (58).

For proper activation of a T cell, a signaling threshold must be overcome (59, 60); too weak a signal and no activation occurs; too strong a signal results in T cells dying by apoptosis (61, 62). Lck is a key tyrosine kinase involved in the phosphorylation of effectors downstream of the TCR. Levels of phosphorylation at an activatory residue (Lck Tyr394) prior to signaling influences downstream signaling strength (32). We observed that equilibration at various oxygen levels for 24 hours altered the number of cells containing active Lck and therefore could potentially influence subsequent signaling strength and possibly explain the decrease in proliferation observed at both extremes of oxygen exposure. Lck has previously been linked to T cell response to hypoxia: Lck forms a complex with the potassium channel Kv1.3 (63) and Kv1.3 channels are sensitive to oxygen levels but only in the presence of Lck (64). Therefore, the reduction in Lck activity we observed after 24 hours equilibration under hypoxia may result in downregulation of proliferation by reducing the calcium signaling capabilities of the cell post-TCR stimulation.

CD69 is a leukocyte early activation marker that has been found to inhibit leukocyte egress from organs and tissue via the internalisation of SIP1 (65, 66). In our *in vitro* system we observed increasing CD69 expression as oxygen levels decreased. CD69 positive cells are rarely observed in circulation (67), and are instead found in inflammatory environments including chronically inflamed tissues (68, 69). Furthermore, CD69 expression is associated with tissue-resident memory cells (TRM) (34, 67, 70, 71). Labiano et al. described an increase in CD69 expression in PBMCs stimulated by anti-CD3 at 1% oxygen and found that the *cd69* promoter contains a HIF-1α response element (10, 72). At 8.5% oxygen, where HIF-1α is not thought to be stabilised long-term, we observed the lowest expression of CD69, whereas an intermediate oxygen level of 3%, where HIF-1α is thought to be stabilised (56), revealed an intermediate level of CD69, highlighting that CD69 expression may reflect the lack of oxygen in its environment, and hence why on this occasion stimulating at atmospheric oxygen gave a pattern more similar to physiologically healthy oxygen levels.

Oxygen level, therefore, may play a regulatory role in the adaptive immune system, and needs to be considered when investigating lymphocyte behaviour and function. A vast wealth of studies has contributed to our understanding CD4+ T cell biology in hypoxia by comparing 1-2% oxygen to just 21% oxygen only (9, 35, 44–48, 57, 73–77). However, the use of 21% oxygen may be inappropriate and skew results away from what occurs *in vivo*.

In the wider scientific community, it is now increasingly accepted that supraphysiologic oxygen conditions are inappropriate and pro-oxidant in nature (78). Primary cell senescence in hyperoxic conditions is now well established (79) with adaption to increasingly oxidative environments evident in cell lines cultured in hyperoxic conditions (80). Endothelial cells cultured at 5% and 21% oxygen differed in gene expression downstream of Nrf2, highlighting that hyperoxic *in vitro* work may artificially generate gene expression signatures (31). Relevant to this study, T cell differentiation from CD34+ progenitors was improved when cultured at 5% oxygen compared to 21% oxygen (81).

Often, the term ‘normoxia’ is used for culture at 21% oxygen, but we would like to challenge that from the perspective of an *ex vivo* cell, where ‘normoxia’ is anything from 0.5-10% oxygen, but never double this. Our observation that resting T cell TCR activation thresholds are influenced by their prior oxygen exposure suggests that T cells migrating into inflamed tissue may be influenced by where they have migrated from. T cells moving into tissue from oxygen-rich blood may be better equipped to proliferate yet may limit their cytokine production. In comparison those coming from oxygen-deplete environments, or being tissue-resident in nature, may have reduced proliferation and a more pro-inflammatory cytokine signature. It also highlights how equilibration of cells to desired oxygen levels is prudent, especially in the light of recent progress in the therapeutic use of T cells. This is also pertinent to *in vitro* migration assays where incorrect oxygen levels may skew or even dampen responses.

We observed that cells behaved differently, at different timepoints, at different oxygen levels. When placed into the perspective of a T cell that has just migrated into an inflamed tissue, or one that has become exhausted in a more chronic inflammatory environment, this is of importance. Therefore, we encourage researchers to consider the oxygen environment of their target tissue when completing *in vitro* research, with regards to both health and disease, to further the therapeutic potential and understanding of T cells. Considerations need to include equilibration to relevant oxygen levels for both cells and media, and the maintenance of oxygen levels over the course of an experiment including during media changes.

## Data Availability

The raw data supporting the conclusions of this article will be made available by the authors, without undue reservation.
