# Supplementary material for: Deviation from physiologically appropriate oxygen levels alters proliferation, cytokine production and proximal antigen receptor signalling in CD4+ memory T cells
Source: Front Immunol. 2026 May 26;17:1833034. doi: 10.3389/fimmu.2026.1833034 (PMC13246643; doi:10.3389/fimmu.2026.1833034)
Supplement: Supplementary file 1 [file Table1.docx]

**Supplementary methods.**

**Treatment of CD4+ memory T cells with *N*-acetyl cysteine**. For treatment with *N*-acetyl-*L*-cysteine (NAC), cells were placed directly in medium containing 1mM NAC and equilibrated to the indicated oxygen level. NAC was purchased from Sigma-Aldrich (St. Louis, MO, USA). Cells were plated and stimulated as described in the main methods section of the manuscript. Stimulation occurred after 24 hours equilibration to the desired oxygen level.
