# Supplementary material for: Deviation from physiologically appropriate oxygen levels alters proliferation, cytokine production and proximal antigen receptor signalling in CD4+ memory T cells
Source: Front Immunol. 2026 May 26;17:1833034. doi: 10.3389/fimmu.2026.1833034 (PMC13246643; doi:10.3389/fimmu.2026.1833034)
Supplement: Supplementary file 2 [file DataSheet1.pdf]

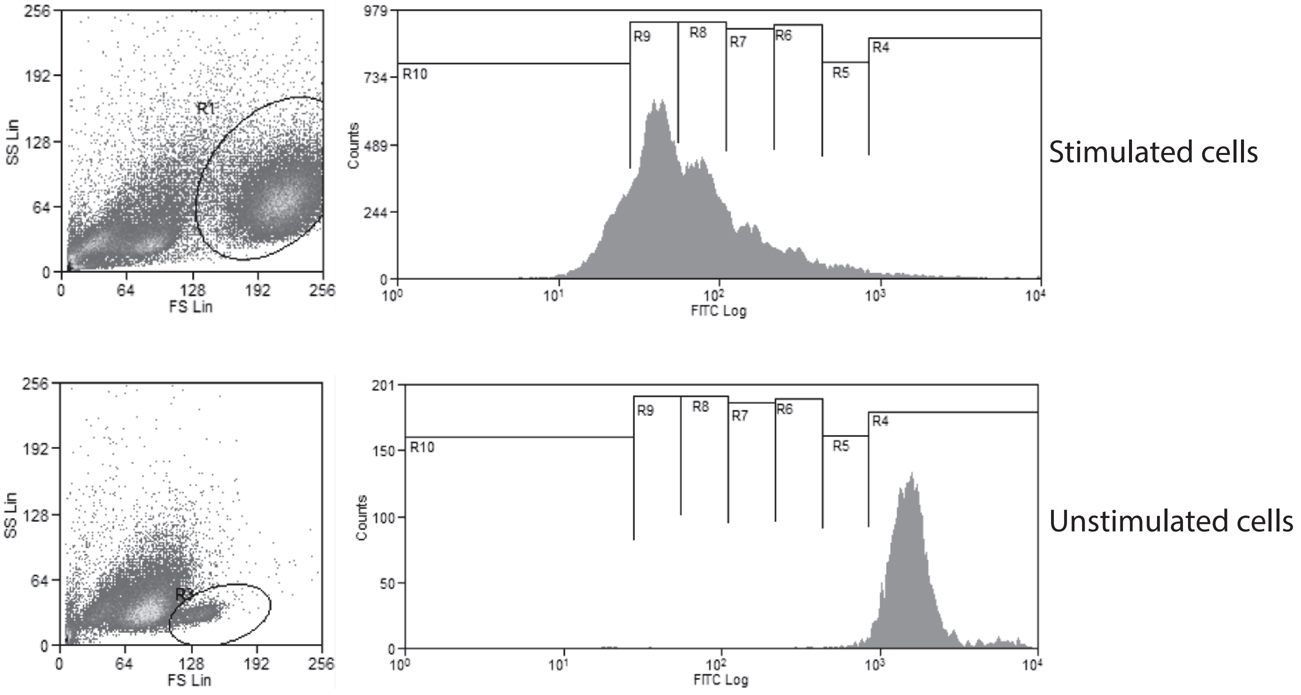

Supplementary Figure 1. Gating strategy for stimulated and unstimulated cells, with example CFSE plots for each. Cells were equilibrated to the stated oxygen level for 24 hours and then stimulated with anti-CD3/CD28. Left hand panels show example forward/side scatter flow cytometry plots with gating strategy for each, pertinent to all stimulation strategies. Right hand plots show gates applied for proliferation analysis for cells stained with 1μM CFSE. Proliferation was assessed after four days stimulation by flow cytometry.
