# Supplementary material for: Deviation from physiologically appropriate oxygen levels alters proliferation, cytokine production and proximal antigen receptor signalling in CD4+ memory T cells
Source: Front Immunol. 2026 May 26;17:1833034. doi: 10.3389/fimmu.2026.1833034 (PMC13246643; doi:10.3389/fimmu.2026.1833034)
Supplement: Supplementary file 3 [file DataSheet2.pdf]

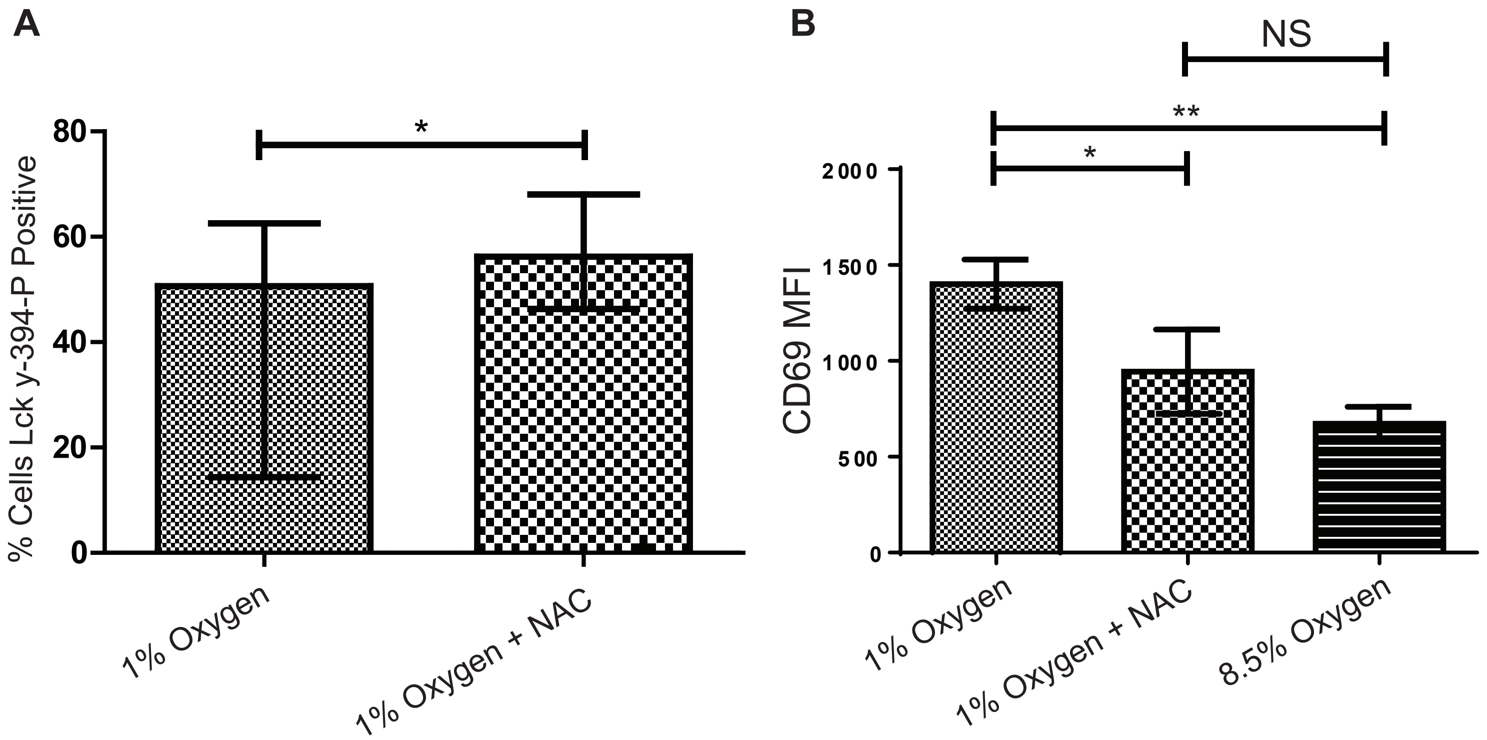


**Supplementary Figure 2**. Treatment with the anti-oxident *N*-acetyl cysteine (NAC) alters CD4+ memory T cell responses to hypoxia. (**A**) CD4+ memory T cells were equilibrated at 1% oxygen with and without NAC treatment for 24 hours before being fixed with 4% paraformaldehyde. Cells (unstimulated) were stained for phosphorylation at Tyr394 on Lck and assessed by flow cytometry. Percentage positive cells were determined by comparison with isotype controls. A Mann Whitney U statistical test was used for the analysis and median values are shown (minimum of 9 donors). * $p<0.05$. (**B**) CD4+ memory T cells were equilibrated for 24 hours at 1% or 8.5% oxygen and treated with NAC where stated. They were then stimulated for 48 hours before being stained for expression of CD69 and assessed by flow cytometry to determine CD69 MFI expression. Statistical analysis was performed using a Paired t-test and mean values are shown. * $p< 0.05$. **$p< 0.005$. NS non-significant. Five matched donors were run for each treatment.
